# Optimum flux rerouting for efficient production of naringenin from acetate in engineered *Escherichia coli*

**DOI:** 10.1186/s13068-022-02188-w

**Published:** 2022-09-02

**Authors:** Dong Hwan Kim, Hyun Gyu Hwang, Gyoo Yeol Jung

**Affiliations:** 1 Department of Chemical Engineering, Pohang University of Science and Technology, 77 Cheongam-Ro, Nam-Gu, Pohang, 37673 Gyeongbuk Korea; 2 Institute of Environmental and Energy Technology, Pohang University of Science and Technology, 77 Cheongam-Ro, Nam-Gu, Pohang, 37673 Gyeongbuk Korea; 3 School of Interdisciplinary Bioscience and Bioengineering, Pohang University of Science and Technology, 77 Cheongam-Ro, Nam-Gu, Pohang, 37673 Gyeongbuk Korea

**Keywords:** Flavonoids, Acetate, Glyoxylate cycle, Phosphoenolpyruvate carboxykinase, Metabolic engineering

## Abstract

**Background:**

Microbial production of naringenin has received much attention owing to its pharmaceutical applicability and potential as a key molecular scaffold for various flavonoids. In the microbial fermentation, a cheap and abundant feedstock is required to achieve an economically feasible bioprocess. From this perspective, utilizing acetate for naringenin production could be an effective strategy, with the advantages of both low-cost and abundant feedstock. For the efficient production of naringenin using acetate, identification of the appropriate regulatory node of carbon flux in the biosynthesis of naringenin from acetate would be important. While acetyl-CoA is a key precursor for naringenin production, carbon flux between the TCA cycle and anaplerosis is effectively regulated at the isocitrate node through glyoxylate shunt in acetate metabolism. Accordingly, appropriate rerouting of TCA cycle intermediates from anaplerosis into naringenin biosynthesis via acetyl-CoA replenishment would be required.

**Results:**

This study identified the isocitrate and oxaloacetate (OAA) nodes as key regulatory nodes for the naringenin production using acetate. Precise rerouting at the OAA node for enhanced acetyl-CoA was conducted, avoiding extensive loss of OAA by fine-tuning the expression of *pckA* (encoding phosphoenolpyruvate carboxykinase) with flux redistribution between naringenin biosynthesis and cell growth at the isocitrate node. Consequently, the flux-optimized strain exhibited a significant increase in naringenin production, a 27.2-fold increase (with a 38.3-fold increase of naringenin yield on acetate) over that by the unoptimized strain, producing 97.02 mg/L naringenin with 21.02 mg naringenin/g acetate, which is a competitive result against those in previous studies on conventional substrates, such as glucose.

**Conclusions:**

Collectively, we demonstrated efficient flux rerouting for maximum naringenin production from acetate in *E. coli*. This study was the first attempt of naringenin production from acetate and suggested the potential of biosynthesis of various flavonoids derived from naringenin using acetate.

**Supplementary Information:**

The online version contains supplementary material available at 10.1186/s13068-022-02188-w.

## Background

Naringenin, a secondary metabolite that can be obtained from natural plants, is a value-added chemical with high pharmaceutical applicability, such as oxygen radical elimination, and anti-inflammatory and antiviral properties [1–3]. In addition, it has value as a key scaffold molecule for the biosynthesis of various flavonoids [4, 5]. Traditionally, naringenin is extracted from plants; however, the conventional method has been limited by low yield from natural sources, complicated purification involved in a large number of solvents, and scalability issues [5–7]. Accordingly, attempts have been made to produce naringenin by microbial biosynthesis via heterologous production in *Escherichia coli* and *Saccharomyces cerevisiae* with advances in metabolic engineering and synthetic biology. For microbial production of naringenin, low-cost and abundant feedstock would be required to achieve economically feasible and sustainable bioprocesses. In this regard, acetate has emerged as a promising carbon source, showing high potential for industrial use, since it can plentifully be obtained from inexpensive natural sources, such as lignocellulose biomass and carbon dioxide, at reasonable costs [8–10]. Furthermore, acetate is an eco-friendly renewable resource that can be produced from fermentation of industrial by-products and syngas [11, 12]. Therefore, utilizing acetate for microbial naringenin production would be an effective strategy for the construction of economically viable bioprocesses and reducing the cost of substrates.

**Fig. 1:**
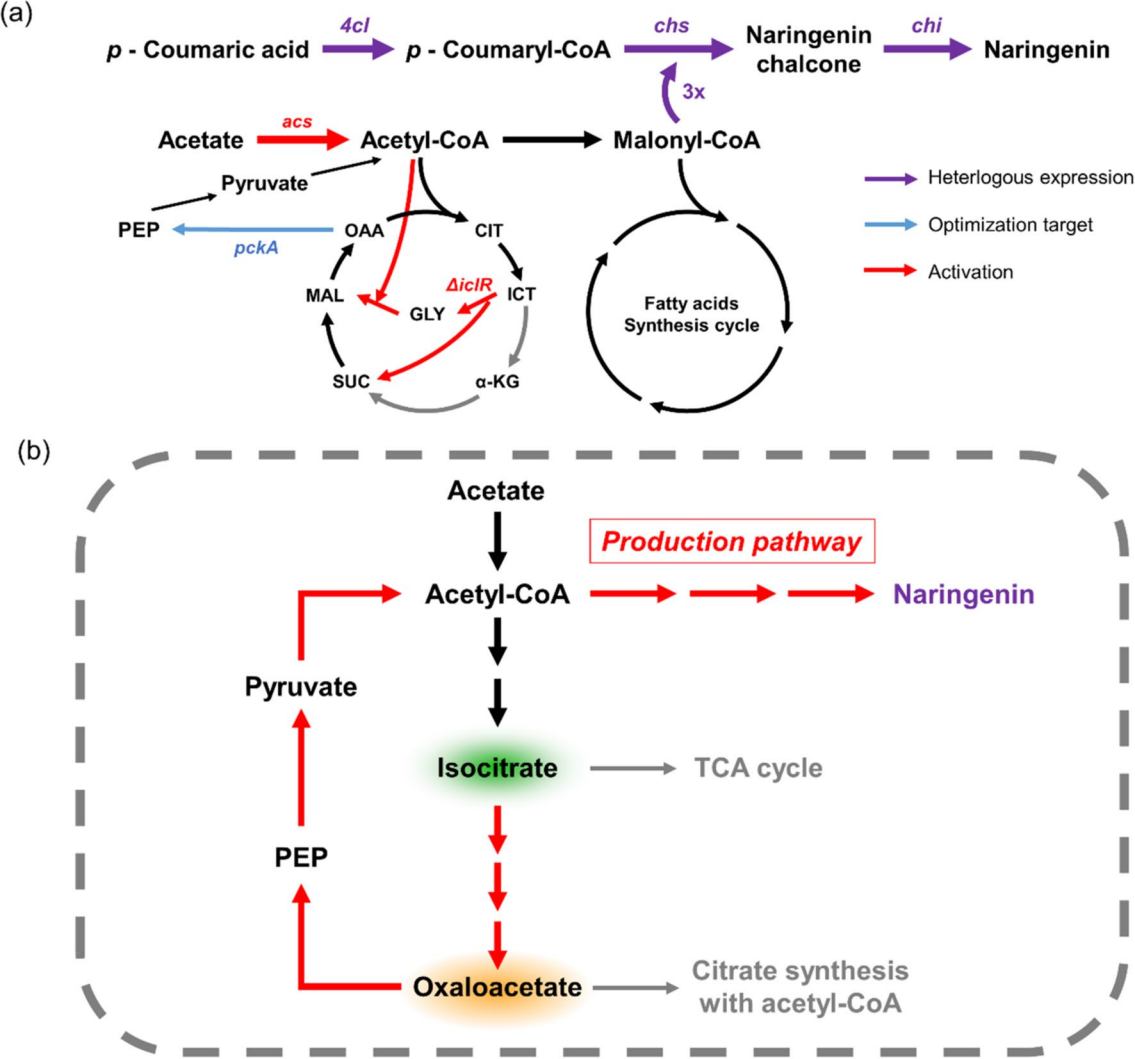
Overall metabolic engineering strategies used for naringenin production from acetate and a schematic diagram. *PEP* phosphoenolpyruvate, *CIT* citrate, *ICT* isocitrate, *α-KG* α-ketoglutarate, *SUC* succinate, *MAL* malate, *OAA* oxaloacetate, *GLY* glyoxylate, *TCA cycle* tricarboxylic acid cycle

To achieve the efficient bioconversion of acetate to naringenin, enhancement of naringenin biosynthetic pathway through malonyl-CoA supply from improved acetyl-CoA availability would be necessary [13–15] (Fig. 1). Indeed, downregulation of the *gltA* gene, encoding citrate synthase, had previously been attempted for the enhancement of acetyl-CoA availability. However, since citrate synthase is a pace-making enzyme for the flux toward tricarboxylic acid (TCA) cycle, *gltA* repression might cause severe damage to overall cell physiology, leading to critical effects on energy generation and cell building block synthesis, thereby making it an unsuitable engineering target [16, 17]. Hence, selecting appropriate nodes for metabolic flux redistribution between naringenin biosynthesis and cell growth would be important for increased naringenin production. In this context, isocitrate node could be a key that can specifically redistribute the flux between naringenin production and cell growth as a branching point of the glyoxylate and the TCA cycles [18–21]. Furthermore, activating the glyoxylate cycle can facilitate anaplerotic reaction for acetate assimilation and reduce CO_2_ loss from the TCA cycle [21, 22]. In addition to the isocitrate node, it would be important to identify the regulatory node to enable a tight linkage between TCA cycle intermediates via anaplerosis of the glyoxylate cycle and naringenin biosynthesis for enhanced production of naringenin. According to previous studies of acetate-grown *E. coli* metabolism, the global expression profiling and metabolic flux analysis reported that the flux of oxaloacetate to PEP was strengthened under acetate metabolism, which indicated that the gluconeogenic flux of TCA intermediates are mainly distributed at the oxaloacetate (OAA) node [18, 19, 23]. From this perspective, the OAA node could be critical for flux regulation, to efficiently convert OAA, which is mainly accumulated via anaplerotic reaction of the glyoxylate cycle, into phosphoenolpyruvate (PEP) and to reflux it to naringenin biosynthesis (Fig. 1).

For this purpose, upregulating the *pckA* gene, encoding phosphoenolpyruvate carboxykinase, to strengthen carbon flux from OAA to PEP could be an effective engineering target [18, 19]. However, excessive overexpression of *pckA* can cause extensive loss of OAA, leading to significant metabolic imbalance, such as reduction of TCA cycle activity and inhibition of cell building block synthesis, resulting in reduced cell growth and decreased naringenin production. Therefore, precise rebalancing of flux at the OAA node through appropriate levels of *pckA* upregulation would be important for efficient naringenin production. Overall, metabolic flux redistribution via glyoxylate cycle activation and precise optimization of the flux at the OAA–PEP node might be effective in achieving high yield and productivity of naringenin biosynthesis from acetate.

In this study, we activated the anaplerotic reaction of glyoxylate cycle through *iclR* deletion, while enhancing acetyl-CoA availability by the overexpression of *acs* gene encoding acetyl-CoA synthase (Fig. 1). Moreover, the strategy of further boosting metabolic flux at the OAA–PEP node was attempted to facilitate the flux of PEP–pyruvate–acetyl-CoA node from TCA cycle intermediates for increased naringenin production. In particular, precise tuning of *pckA* expression under large and balanced expression ranges was conducted for exploration of proper flux balance at the OAA node. Remarkably, the flux-optimized strain showed significant increase of naringenin production, a 27.2-fold enhancement (with a 38.3-fold increase of naringenin yield on the carbon substrate) over that of the unoptimized strain. Consequently, the optimized strain produced 97.02 mg/L naringenin with a yield of 21.02 mg naringenin/g acetate (the highest naringenin yield among carbon substrates) under optimized cultivation conditions, thereby achieving a competitive result against those from previous studies on conventional substrates (Additional file 1: Table S1). Collectively, this study was the first attempt to establish naringenin biosynthesis from acetate through microbial processes. Naringenin production was especially drastically improved by the activation of the glyoxylate cycle and enhancement of the naringenin biosynthetic pathway through fine-tuning of the flux at the OAA–PEP node, thereby demonstrating its potential as a key strategy of metabolic engineering for the biosynthesis of various flavonoids derived from naringenin using acetate.

## Results and discussion

### Construction of a naringenin-producing strain using acetate

For naringenin biosynthesis from acetate in *E. coli*, three heterologous enzymes needed to be introduced into *E. coli*: 4-coumaroyl-CoA ligase (4CL) which catalyzes the formation of *p*-coumaryl-CoA ester involving 1 mol of ATP, Coenzyme A and *p*-coumaric acid; chalcone synthase (CHS) which catalyzes the condensation to naringenin chalcone using 3 mol of maloyl-CoA and 1 mol of *p*-coumaryl-CoA; chalcone isomerase (CHI) which catalyzes the stereospecific cyclization of naringenin chalcone to naringenin (Fig. 1). In particular, we took advantage of a homologous enzyme combination, which had been validated for the highest naringenin production in a previous study, to introduce the optimized naringenin biosynthetic pathway; the combination included 4CL from *Arabidopsis thaliana*, CHS from *Petunia hybrida*, and CHI from *Citrus maxima* [24]. In addition, expression of the three genes was optimized at transcriptional level using promoter variants with diverse strengths. Therefore, we designed a base strain, BN, for producing naringenin from acetate based on the optimized naringenin biosynthetic pathway [24].

**Fig. 2:**
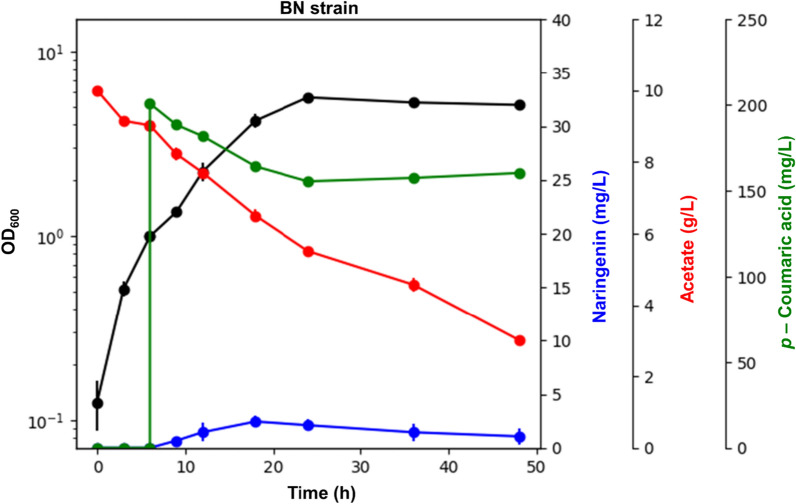
Fermentation profiles of BN strain (engineered *E. coli* to produce naringenin from acetate). BN strain refers to *Escherichia coli* BL21 Star™(DE3) with heterologous expression of essential enzymes for naringenin production, namely, 4CL, CHS, and CHI. 200 mg/L *p*-coumaric acid and 1 mM IPTG were added when culture broths reached an OD_600_ of 1.0. Flask cultures were performed for 48 h in biological triplicates. Error bars indicate the standard deviations of biological triplicates. *4CL* 4-coumaroyl-CoA ligase, *CHS* chalcone synthase, *CHI* chalcone isomerase, *IPTG* isopropyl β-d-thiogalactopyranoside

We evaluated naringenin production capacity of the constructed strain, harboring the recombinant naringenin biosynthetic pathway, with 10 g/L acetate as the carbon source (Fig. 2). The BN strain produced 2.45 mg/L naringenin over 48 h of cultivation, showing the yield of 1.24 mg naringenin/g acetate. Therefore, heterologous expression of 4CL, CHS, and CHI successfully enabled *E. coli* to produce naringenin from acetate; however, the naringenin concentration was relatively low compared to that reported in previous studies on naringenin production from conventional substrates (Additional file 1: Table S1). Therefore, further strain improvement for enhanced acetyl-CoA availability would be required to achieve efficient production of naringenin from acetate.

### Effect of *acs* overexpression and *iclR* deletion on naringenin production using acetate

**Fig. 3:**
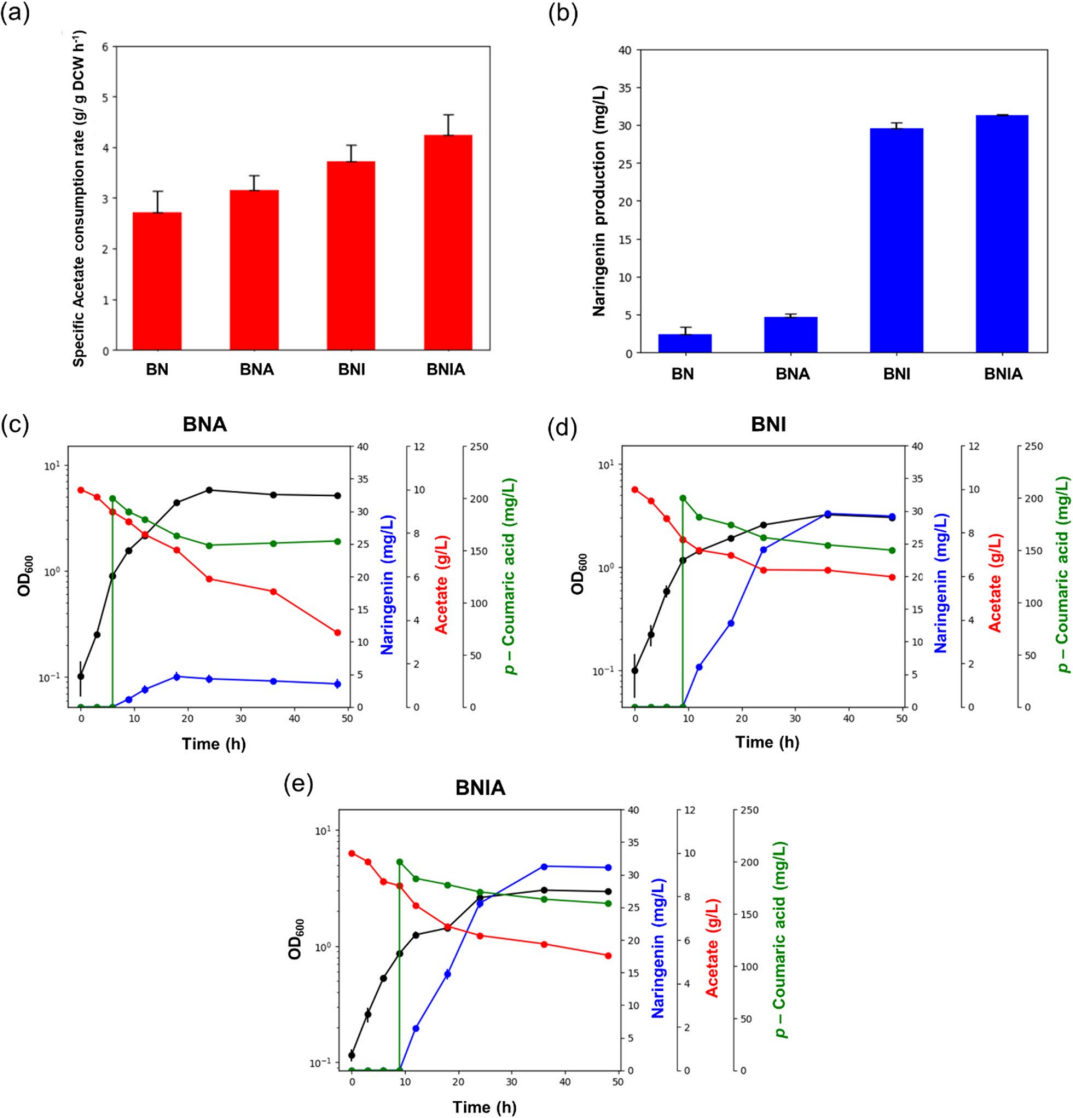
Evaluation of the effect of *acs* overexpression and *iclR* deletion. **a** Specific acetate consumption rate of engineered *E. coli* strains and **b** their maximum naringenin production titer. **c**–**e** Fermentation profile of engineered *E. coli* over 48 h culture. 200 mg/L *p*-coumaric acid and 1 mM IPTG were added when culture broths reached an OD_600_ of 1.0. Flask cultures were performed in biological triplicates. Error bars indicate the standard deviations of biological triplicates. BN strain refers *Escherichia coli* BL21 Star™(DE3) with heterologous expression of essential enzymes for naringenin production; *BNA* BN strain with *acs* overexpression, *BNI* BN strain with *iclR* knockout, *BNIA* BN strain with both *acs* overexpression and *iclR* knockout, *IPTG* isopropyl β-d-thiogalactopyranoside

To enhance naringenin production, facilitation of the naringenin biosynthetic pathway via efficient supply of malonyl-CoA from acetyl CoA would be highly important (Fig. 1). Especially, improvement of the availability of acetyl-CoA, an exclusive precursor of malonyl-CoA, was previously suggested to increase naringenin production [13–15, 24]. For enhancing acetyl-CoA availability, first, we accelerated the reaction to convert acetate to acetyl-CoA through overexpression of *acs* gene encoding acetyl-CoA synthase, which was obtained from the genomic DNA of *E. coli* BL21 Star™ (DE3), thereby yielding the BNA strain. Naringenin production by the BNA strain was 82% higher than that by the BN strain, and specific acetate consumption rate of BNA showed a 15% improvement over that of BN (Fig. 3a, b).

In addition, the identification of key regulatory node between cell growth and naringenin biosynthesis would be important. Isocitrate node could be an effective engineering target that can specifically redistribute the flux, as a branching node, between the glyoxylate and TCA cycles [18, 19]. From this perspective, we activated the anaplerotic reaction of glyoxylate cycle and rebalanced the carbon flux at the isocitrate node by deleting the *iclR* gene encoding isocitrate lyase repressor, thereby resulting in the BNI strain. This strain showed a 10.2-fold higher naringenin concentration than the BN strain, with a 38% increase of the specific acetate consumption rate over that of the BN strain (Fig. 3a, b). Compared BNI with BNA, we speculated that the generated acetyl-CoA was mainly used in TCA cycle for cell growth in the BNA, despite the conversion of acetate to acetyl-CoA through *acs* overexpression, which caused relatively low boost of naringenin production in the BNA. From this perspective, we demonstrated that flux rebalancing at the isocitrate node between cell growth and naringenin production was significant for the efficient naringenin biosynthesis.

Furthermore, we evaluated the synergistic effect of *acs* overexpression and *iclR* deletion through the generation of BNIA. The constructed BNIA produced 32.81 mg/L naringenin, an 11.8-fold improvement of naringenin production over that of the BN strain, while its specific acetate consumption rate was 53% higher than that of the BN strain (Fig. 3a–e). Our results showed that activation of the glyoxylate shunt pathway by *iclR* deletion enhanced the supply of TCA cycle intermediates, such as OAA, thereby efficiently facilitating the flux of OAA–PEP–acetyl-CoA for the naringenin biosynthetic pathway. Indeed, previous studies had suggested that facilitation of glyoxylate cycle increased the flux of OAA to PEP, strengthening the replenishment of acetyl-CoA [13, 18, 19, 25]. Therefore, we expected an additional room for flux optimization at the OAA node, in addition to *iclR* deletion considering the importance of a fine-tuned and tight linkage between TCA cycle intermediates via anaplerotic reaction of the glyoxylate cycle and naringenin biosynthesis for increased production of naringenin.

### Enhancement of flux at the OAA to PEP node via *pckA* overexpression

The rewiring strategy to replenish acetyl-CoA from TCA cycle intermediates was highly effective in activating naringenin biosynthesis. Accordingly, we expected that further engineering to strengthen the carbon flux from OAA to PEP would facilitate the naringenin biosynthetic pathway by enhancing acetyl-CoA availability. In this context, we attempted upregulating the *pckA* gene encoding phosphoenolpyruvate carboxykinase for efficient flux redistribution at the OAA node. However, excessive overexpression of *pckA* could lead to extensive OAA loss, which could cause metabolic imbalance, such as inhibition of TCA cycle and insufficient supply of cell building blocks, thereby decreasing naringenin production. Therefore, optimization of flux redistribution at the OAA node through precise upregulation of *pckA* at appropriate levels would be important.

For precise flux rebalancing, we obtained *pckA* gene from the genomic DNA of *E. coli* BL21 Star™ (DE3) and conducted the fine-tuning of *pckA* gene expression at the transcriptional level. To be specific, we generated a wide and balanced variation of expression using various promoters with different promoter strengths, namely, BBa_J23103, BBa_J23113, BBa_J23109, BBa_J23115, BBa_J23106, BBa_J23104, and BBa_J23100 (from weak to strong, in order). We speculated that there would be an optimal point of *pckA* expression to enhance the naringenin biosynthetic pathway by facilitating the acetyl-CoA availability while preventing extensive OAA loss over the wide range of gene expression.

**Fig. 4:**
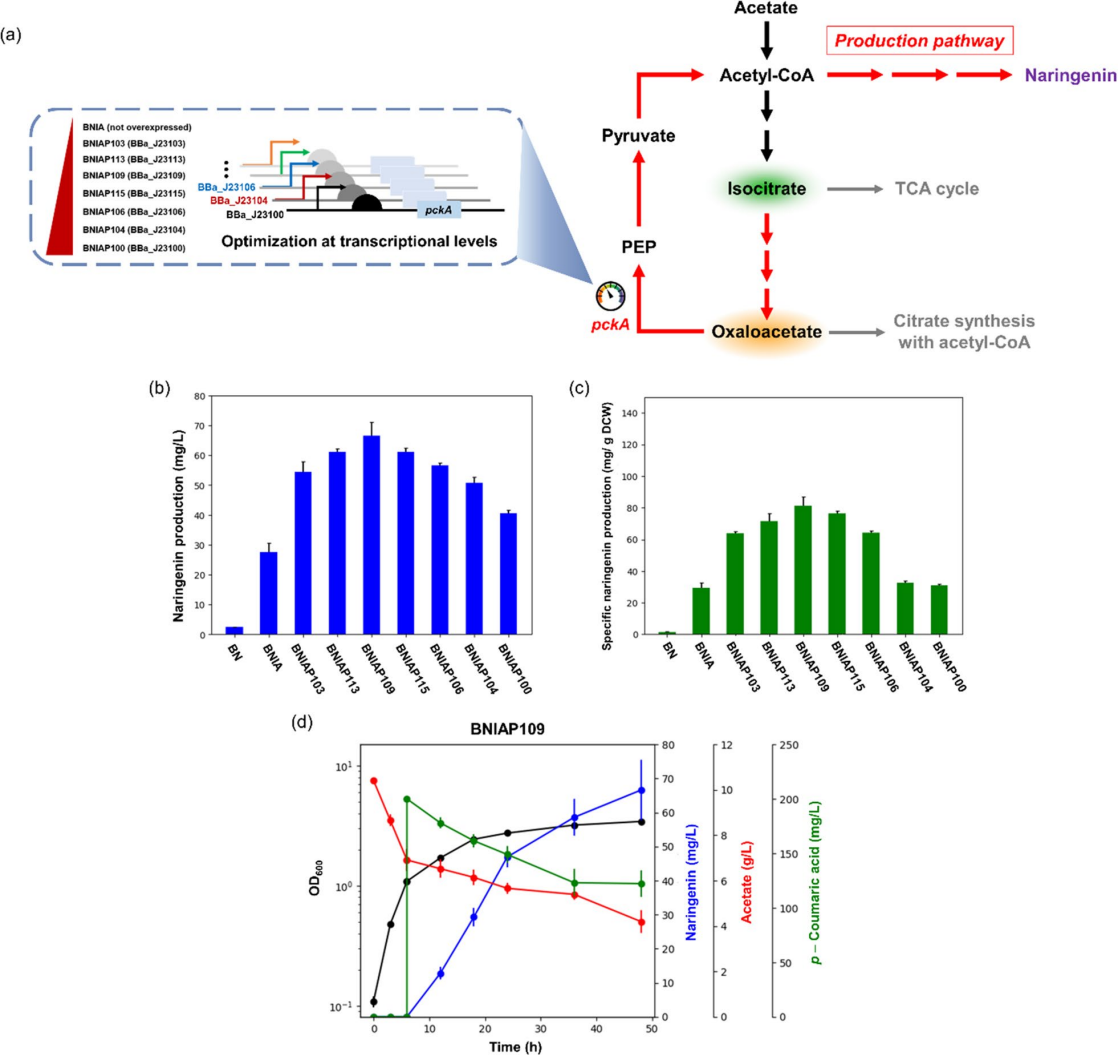
Effect of fine-tuned expression of *pckA* under promoter variants. **a** Schematic diagram of *pckA* expression control using promoter variants with different strengths. **b** Naringenin titer and **c** specific naringenin production by engineered *E. coli* strains with different transcriptional levels of *pckA.* **d** Time-course culture profile of *pckA*-upregulated *E. coli*, the BNIAP109 strain. 200 mg/L *p*-coumaric acid and 1 mM IPTG were added when culture broths reached an OD_600_ of 1.0. Flask cultures were performed for 48 h in biological triplicates. Error bars indicate the standard deviations of biological triplicates. BN strain refers *Escherichia coli* BL21 Star™(DE3) with heterologous expression of essential enzymes for naringenin production; BNIA, BN strain with both *acs* overexpression and *iclR* knockout; BNIAP103, BNIAP113, BNIAP109, BNIAP115, BNIAP106, BNIAP104, BNIAP100 refer BNIA strain with *pckA* upregulation under constitutive promoter BBa_J23103, BBa_J23113, BBa_J23109, BBa_J23115, BBa_J23106, BBa_J23104, BBa_J23100, respectively; *PEP* phosphoenolpyruvate, *TCA cycle* tricarboxylic acid cycle, *IPTG* isopropyl β-d-thiogalactopyranoside

Upon verifying the naringenin production by promoter variants, naringenin concentrations of all variants were found to be increased compared to that of BNIA, where *pckA* expression was not overexpressed (Fig. 4b, c). The result demonstrated that further amplification of acetyl-CoA from TCA cycle intermediates could effectively improve acetyl-CoA supply for naringenin biosynthesis. Notably, enormous increase of naringenin production was observed in the BNIAP109 strain with 66.59 mg/L naringenin and specific production of 81.48 mg/g DCW, which is a 27.2-fold and 54.7-fold improvement over those of the BN strain, respectively (Fig. 4b–d). Furthermore, naringenin yield on acetate in the BNIAP109 strain (10.71 mg naringenin/g acetate) was 38.3-fold higher than that in the BN strain (Additional file 1: Table S2), which suggested that the conversion of acetate to naringenin was effectively improved through precise flux redistribution. Meanwhile, in the variants with promoters stronger than BBa_J23109 for *pckA* expression, naringenin production was rather decreased compared to that in the optimized strain BNIAP109 (Fig. 4b and Additional file 1: Table S2). In addition, with the validation of changes in *pckA* expression level under various promoters through enzyme activity assay, we demonstrated that the optimal *pckA* expression level for the efficient production appeared in the BNIAP109 strain with the highest naringenin production (Additional file 1: Fig. S3). The result suggested that fine-tuning of carbon flux at the OAA–PEP node is crucial for naringenin production and successful achievement of optimal expression of *pckA*. Collectively, we demonstrated the importance of precise flux redistribution to enhance the naringenin biosynthetic pathway while avoiding extensive loss of OAA, which enabled an optimal tight linkage between naringenin biosynthesis and TCA cycle intermediates supplied from anaplerosis of the glyoxylate cycle.

### Culture-condition optimization for enhanced naringenin production

We conducted further optimization of culture conditions for the flux-optimized BNIAP109 strain to improve naringenin production. In the naringenin-producing strains used in this study, 4CL, CHS, and CHI, which are the key enzymes for naringenin biosynthesis, were expressed under the T7 promoters using IPTG as an inducer. Optimum induction point and induction level were reported to be associated with the allocation of cellular resources and to be more complicated than simply affecting protein production levels [26–28]. Indeed, due to this complexity, the optimum culture condition was evaluated for naringenin production from conventional substrates, such as glucose and glycerol, emphasizing that optimum induction conditions are specific to a particular system and must be determined experimentally [24, 29].

**Fig. 5:**
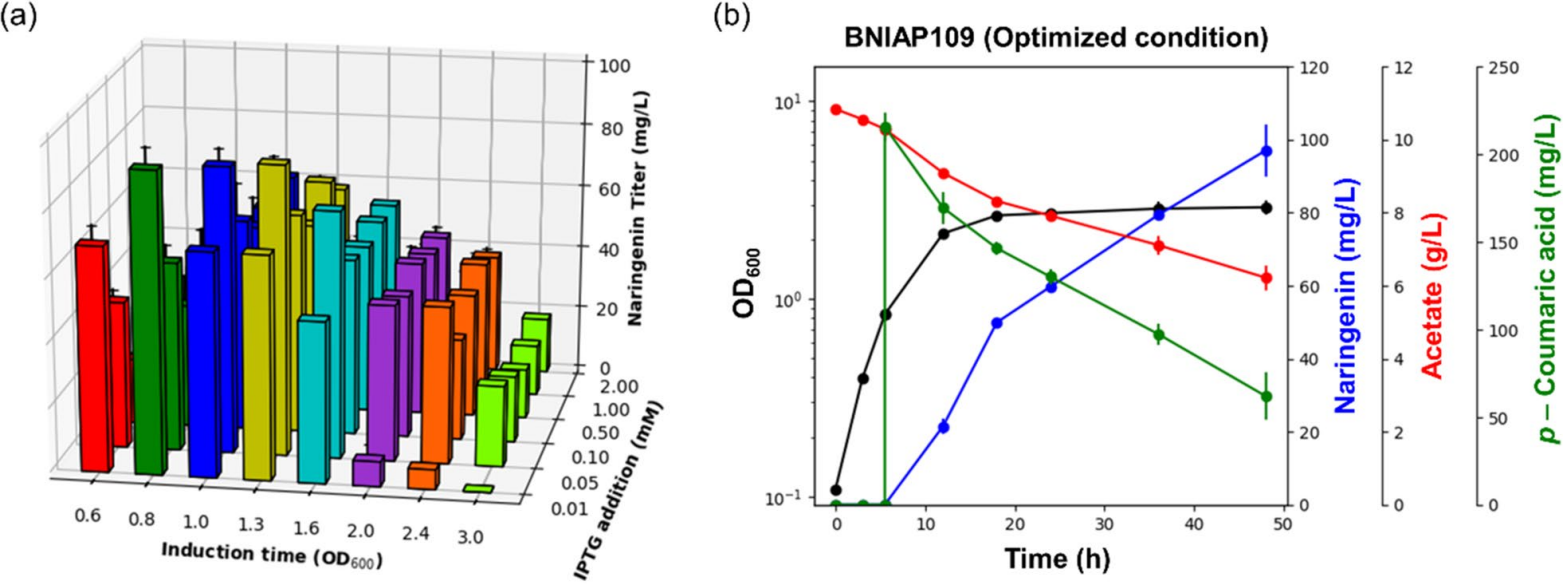
Culture condition optimization of the BNIAP109 strain. **a** Evaluation of naringenin production capacity under different conditions of IPTG concentrations and induction times. **b** Fermentation profile of the BNIAP109 strain under optimized culture conditions, such as induction time at OD_600_ of 0.8 with the addition of 0.01 mM IPTG. Flask cultures were performed for 48 h in biological triplicates. Error bars indicate the standard deviations of biological triplicates. BNIAP109 strain, *Escherichia coli* BL21 Star™(DE3) with heterologous expression of essential enzymes for naringenin production, *acs* overexpression, *iclR* knockout*,* and *pckA* upregulation under constitutive promoter BBa_J23109; *IPTG* isopropyl β-d-thiogalactopyranoside

Consequently, using various induction points, from OD_600_ 0.6 to 3.0_,_ naringenin production capacity of BNIAP109 was found to be notably changed, showing the highest naringenin titer at the induction point of OD_600_ 0.8. In addition to the induction point, under different IPTG concentrations, BNIAP109 showed the highest naringenin production with the addition of 0.01 mM IPTG. As a result, heterologous expression of 4CL, CHS, and CHI for efficient production of naringenin from acetate was optimized at an appropriate concentration of IPTG rather than at high concentrations of IPTG. Remarkably, the highest naringenin titer of 97.02 mg/L with naringenin yield (21.02 mg naringenin/g acetate and 0.631 g naringenin/g *p*-coumaric acid) was observed under the optimized culture conditions, induction time being at OD_600_ 0.8 with the addition of 0.01 mM IPTG (Fig. 5a, b), which achieved competitive result over those of previous studies on conventional substrates (Additional file 1: Table S1).

## Conclusions

In this study, efficient flux rerouting for maximum naringenin production from acetate was demonstrated in *E. coli*. Initially, the naringenin-producing strain using acetate, namely, the BN strain, was constructed through heterologous expression of key enzymes 4CL, CHS, and CHI for naringenin biosynthesis. The BN strain produced 2.45 mg/L naringenin from acetate; however, the naringenin concentration was remarkably low. Accordingly, facilitation of the naringenin biosynthetic pathway was attempted via malonyl-CoA supply arising from enhanced acetyl-CoA availability. For this purpose, the *acs* gene, responsible for the conversion of acetate to acetyl-CoA, was overexpressed, and flux redistribution between cell growth and naringenin biosynthesis was applied at the isocitrate branching node, resulting in the BNIA strain. The BNIA strain showed a 53% higher acetate consumption rate over that of the BN; notably, it produced 32.81 mg/L naringenin (a 11.8-fold increase of naringenin production over that of the BN strain). In addition, we devised the precise rewiring strategy to replenish acetyl-CoA from TCA intermediates by fine-tuning *pckA* expression at transcriptional levels. Surprisingly, the optimized strain, BNIAP109, showed significant increase of naringenin production, a 27.2-fold enhancement (with a 38.3-fold increase of naringenin yield on the carbon substrate) over that of the BN. Consequently, under the optimized culture condition for BNIAP109, 97.02 mg/L naringenin was produced, with 21.02 mg naringenin/g acetate (the highest naringenin yield among carbon substrates), which was a competitive result over those of previous studies using conventional substrates (Additional file 1: Table S1). Taken together, we could successfully demonstrate the high-efficiency production of naringenin from acetate through a precise flux redistribution strategy at the key regulatory nodes and highlighted the importance of fine-tuned and tight linkage between TCA cycle and naringenin biosynthesis and the significance of acetyl-CoA availability for efficient production of naringenin from acetate. This study was the first attempt to produce naringenin using acetate and suggested the potential of the platform strain for production of various flavonoids derived from naringenin using acetate.

## Methods

### Reagents and primers

Plasmid and genomic DNA were purified using GeneAll® Plasmid SV kit and GeneAll® Exgene™ Cell SV kit (GeneAll Biotechnology, Seoul, Korea), respectively. Q5® High-Fidelity DNA Polymerase, restriction endonucleases, and T4 DNA ligase were purchased from New England Biolabs (Ipswich, MA, USA). T4 Polynucleotide Kinase and EmeraldAmp® PCR Master Mix were purchased from Takara Bio Inc. (Shiga, Japan). Oligonucleotides were synthesized by Cosmogenetech (Seoul, Korea) (Additional file 1: Table S3). Other reagents were purchased from Sigma-Aldrich (St. Louis, MO, USA) unless specified.

### Construction of bacterial strains and plasmids

Bacterial strains and plasmids used in this study are listed in Additional file 1: Table S4. *E. coli* Mach1-T1® was used to proliferate all plasmids for cloning experiments, naringenin production was performed in BL21 Star™ (DE3) and its derivatives. Transcriptionally optimized pFlavo^opt^, containing 4-coumaroyl-CoA ligase (4CL), chalcone synthase (CHS), and chalcone isomerase (CHI) for naringenin production, was obtained from a previous study [24], and other genes were derived from the genomic DNA of *E. coli* BL21 Star™ (DE3). Genetic information of constitutive promoters (Anderson promoter series) and terminator (BBa_B1001) was acquired from the Registry of Standard Biological Parts (http://parts.igem.org). 5′-untranslated regions (5′-UTRs, Additional file 1: Table S5) with specific predicted expression levels were designed using the UTR Library Designer [30].

Restriction enzyme digestion and ligation were used for the construction of plasmids pACYCA and pACYCAP115. Specifically, *acs* and *pckA* genes were amplified from the genomic DNA of *E. coli* BL21 Star™ (DE3) via two sequential PCRs to introduce the constitutive promoter and synthetic 5′-UTR. For the insertion of *acs*, the vector fragment was obtained from pACYCduet-1, as a template, using Vector_*acs*_speI_F/Vector_*acs*_notI_R. The purified PCR fragments of *acs* and its vector were digested using SpeI and NotI and assembled, resulting in pACYCA. Similarly, the vector fragment was amplified from pACYCA as a template, using Vector_*pckA*_kpnI_F/Vector_*pckA*_notI_R, for the insertion of *pckA*. The resulting fragment and the *pckA* PCR fragment were digested using KpnI and NotI, and assembled to construct pACYCAP115. Plasmids with promoter variants of *pckA* (pACYCAP104, pACYCAP106, pACYCAP109, pACYCAP113, and pACYCAP103) were constructed through blunt-end cloning using the forward primers with corresponding constitutive promoter sequence (J23104_*pckA*_blunt_F, J23106_*pckA*_blunt_F, J23109_*pckA*_blunt_F, J23113_*pckA*_blunt_F, and J23103_*pckA*_blunt_F, respectively) and the reverse primer (*pckA*_blunt_R). Deletion of chromosomal *iclR* gene was conducted by the Lambda-Red recombination method using the plasmids pKD46 and pCP20 [31].

### Culture conditions for naringenin production

*E. coli* strains were cultivated in Andrew’s Magic Medium (AMM) containing 100 mL of 10 × MOPS mixture [32], 5.0 g/L K_2_HPO_4_, 3.5 g/L KH_2_PO_4_, 3.5 g/L (NH_4_)_2_HPO_4_, 2 g/L casamino acid, 0.1 mL of 5 g/L thiamine–HCl, and 0.1 mL of 1 M CaCl_2_ supplemented with 10 g/L NaOH-neutralized acetate (pH 7.0) as a carbon source. Antibiotics (100 mg/L ampicillin and 34 mg/L chloramphenicol) were added to the medium for plasmid maintenance.

For naringenin production, single colonies of each strain were inoculated in 15-mL test tube containing 3 mL fresh AMM and incubated overnight at 37 ℃ with continuous shaking (200 rpm). Thereafter, saturated broths were inoculated in 300-mL Erlenmeyer flasks containing 25 mL fresh medium, at an OD_600_ of 0.1, and incubated to reach OD_600_ of 1.0. Refreshed culture broths were re-inoculated into 25 mL fresh medium with OD_600_ of 0.1 and incubated at a culture temperature of 37 ℃, with agitation at 200 rpm. When culture broths reached an OD_600_ of 1.0, 200 mg/L *p*-coumaric acid and 1 mM isopropyl β-d-thiogalactopyranoside (IPTG) for the induction of *4cl*, *chs*, and *chi* genes were added to the broth, lowering the temperature to 30 ℃ after induction. All experiments were performed in biological triplicates. OD_600_ of the broths was recorded using a UV-1700 spectrophotometer (Shimadzu, Kyoto, Japan) and the pH was adjusted to 6.8–7.1, with a 10 M HCl solution, using an Orion™ 8103BN ROSS™ pH meter (Thermo Fisher Scientific). Culture samples were periodically collected and stored at -80 ℃ for further analysis.

### Enzyme activity assay

The promoter strength variants were cultivated until each variant had the same OD_600_ value, then the cell pellets were harvested by centrifugation at 15,814 × g for 10 min at 4 °C. Cell lysates were prepared through the addition of 0.1 mM phosphate buffer solution (pH 8.0) to the cell pellets and disruption by the sonication (Q125 Sonicator, Qsonica, CT, USA). Enzyme activity was assayed according to the previous study with minor modifications [33]. The reaction mixture containing 4 mM ATP, 4 mM MgCl_2_, 4 mM oxaloacetate, and 0.1 mM phosphate buffer solution were added to cell lysates up to 180 µl, then enzyme reactions were performed for 60 min at 30 °C. The consumption of oxaloacetate were measured using an Aminex HPX-87H column (Bio-Rad Laboratories, Richmond, CA, USA) and Shodex RI-101 detector (Shodex, Klokkerfaldet, Denmark), using 5 mM H_2_SO_4_ as the mobile phase at a flow rate of 0.6 mL/min at 14 ℃ [33].

### Metabolites analysis

Ultimate 3000 high-performance liquid chromatography system (Dionex, Sunnyvale, CA, USA) was used to analyze the metabolites contained in culture broths. For the detection of naringenin production and *p*-coumaric acid consumption, the culture broth and an equal amount of absolute ethanol were mixed and centrifuged for 10 min at 13,000 rpm. Thereafter, the supernatant was analyzed with an Acclaim 120 C18 column (Dionex) and a UV–Vis diode array detector. Acetonitrile and water, each containing 0.1% formic acid, were used as the mobile phase, at a flow rate of 1 mL/min, using the following multi-gradient flow program: 10–40% acetonitrile for 0–10 min and 40–60% acetonitrile for 10–15 min with absorbance detection at 280 nm. Acetate consumption was identified with Aminex HPX-87H column (Bio-Rad Laboratories, Richmond, CA, USA) and Shodex RI-101 detector (Shodex, Klokkerfaldet, Denmark), using 5 mM H_2_SO_4_ as the mobile phase at a flow rate of 0.6 mL/min at 14 ℃.

## Supplementary Information

**Additional file 1.** It contains supplementary data associated with this article. **Table S1**. Previous studies on naringenin biosynthesis in similar cultivation conditions. **Table S2**. Naringenin production performance of bacterial strains used in this study. **Table S3**. Oligonucleotides used in this study. **Table S4**. Bacterial strains and plasmids used in this study. **Table S5**. Synthetic 5′ untranslated region sequences and their predicted expression levels. **Fig. S1**. Time-course culture profiles of *pckA* upregulated strain. **Fig. S2**. Fermentation condition optimization of the BNIAP109 strain in variance of induction time and IPTG concentration. **Fig. S3**. Normalized expression level of *pckA* under various promoter strengths.

## Data Availability

All data generated or analyzed during this study are included in this published article and its supplementary information files.

## Abbreviations

*TCA cycle*: Tricarboxylic acid cycle
*OAA*: Oxaloacetate
*PEP*: Phosphoenolpyruvate
*E. coli*: *Escherichia coli*
*4CL*: 4-Coumaroyl-CoA ligase
*CHS*: Chalcone synthase
*CHI*: Chalcone isomerase
*PCK*: Phosphoenolpyruvate carboxykinase
*IPTG*: Isopropyl β-d-thiogalactopyranoside
*5′﻿-UTRs*: 5′-Untranslated regions
*DCW*: Dry cell weight
