# Supplementary material for: Optimum flux rerouting for efficient production of naringenin from acetate in engineered Escherichia coli
Source: Biotechnol Biofuels Bioprod. 2022 Sep 2;15:90. doi: 10.1186/s13068-022-02188-w (PMC9440541; doi:10.1186/s13068-022-02188-w)
Supplement: Supplementary file 1 — Additional file 1. It contains supplementary data associated with this article. Table S1. Previous studies on naringenin biosynthesis in similar cultivation conditions. Table S2. Naringenin production performance of bacterial strains used in this study. Table S3. Oligonucleotides used in this study. Table S4. Bacterial strains and plasmids used in this study. Table S5. Synthetic 5′ untranslated region sequences and their predicted expression levels. Fig. S1. Time-course culture profiles of pckA upregulated strain. Fig. S2. Fermentation condition optimization of the BNIAP109 strain in variance of induction time and IPTG concentration. Fig. S3. Normalized expression level of pckA under various promoter strengths. [file 13068_2022_2188_MOESM1_ESM.docx]

Supporting Information for:

**Optimum flux rerouting for efficient production of naringenin from acetate in engineered *Escherichia coli***


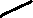


Dong Hwan Kim^a,1^, Hyun Gyu Hwang^b,1^, Gyoo Yeol Jung^a,c,*^

^a^ Department of Chemical Engineering, Pohang University of Science and Technology, 77 Cheongam-Ro, Nam-Gu, Pohang, Gyeongbuk 37673, Korea

^b^ Institute of Environmental and Energy Technology, Pohang University of Science and Technology, 77 Cheongam-Ro, Nam-Gu, Pohang, Gyeongbuk 37673, Korea

^c^ School of Interdisciplinary Bioscience and Bioengineering, Pohang University of Science and Technology, 77 Cheongam-Ro, Nam-Gu, Pohang, Gyeongbuk 37673, Korea.

^*^Correspondence to:

(Gyoo Yeol Jung)

Mailing address: Department of Chemical Engineering, Pohang University of Science and Technology, 77 Cheongam-Ro, Nam-Gu, Pohang, Gyeongbuk 37673, Korea

E-mail: [gyjung@postech.ac.kr](mailto:gyjung@postech.ac.kr)

^1^ Dong Hwan Kim and Hyun Gyu Hwang contributed equally to this work.

**Contents**

Table S1 – S5

Fig. S1 – S3

**Supplementary tables**

**Table S1.** Previous studies on naringenin biosynthesis in similar cultivation conditions.

| **Microorganism** | **Substrate** | **Strategy** | **Fermentation type** | **Titer** | **Yield^*^** | **Reference** |
| --- | --- | --- | --- | --- | --- | --- |
| *E. coli* | Acetate, *p*-coumaric acid | Precise rebalancing of flux at critical regulatory nodes for naringenin production from acetate | Batch | 97.02 mg/L | 1.99% | This study |
| *E. coli* | Glucose, *p*-coumaric acid | Transcriptional optimization of 4CL, CHS, and CHI through engineered vectors | Batch | 36.7 mg/L | NA | [1] |
| *S. cerevisiae* | Glucose | Introduction of naringenin biosynthesis genes from *A. thaliana* and construction of *de novo* pathway for naringenin production form sole carbon source | Batch | 40.3 mg/L | 0.20% | [2] |
| *S. cerevisiae* | Glucose, *p*-coumaric acid | Transcriptional optimization of 4CL, CHS, and CHI | Batch | 52 mg/L | NA | [3] |
| *E. coli* | Glycerol, *p*-coumaric acid | Homolog combinations of 4CL, CHS, and CHI and transcriptional optimization | Batch | 40.7 mg/L | 0.20% | [4] |
| *E. coli* | Glycerol, *p*-coumaric acid | Multi-level optimization of 4CL, CHS, and CHI at both transcriptional and translational levels using riboswitch-guided high-throughput screening | Batch | 93.01 mg/L | 0.75% | [5] |
| *E. coli* | Glycerol, *p*-coumaric acid | Enzyme homolog combinations and feeding exogenous *p*-coumaric acid | Batch | 20.8 mg/L | NA | [6] |
| *E. coli* | Glycerol, *p*-coumaric acid | Down-regulation of fatty acid biosynthesis with antisense RNAs | Batch | 91.31 mg/L | NA | [7] |
| *E. coli* | Glucose, *p*-coumaric acid | Increasing acetyl-CoA and malonyl-CoA availability (biotransformation) | Batch | 119 mg/L | NA | [8] |
| *E. coli* | Glucose, *p*-coumaric acid | *de novo* naringenin biosynthesis through *E. coli-E. coli* co-culture | Batch | 41.5 mg/L | NA | [9] |
| *E. coli* | Glucose,  tyrosine | Introduction of genetic circuits to enhance malonyl-CoA availability through the inhibition of fatty acid synthesis | Batch | 126 mg/L | NA | [10] |
| *E. coli* | Glucose, tyrosine | Optimization of expression of 4CL, CHS, and CHI in transcription level (malonate added) | Batch | 191 mg/L | NA | [11] |
| *E. coli, S. cerevisiae* | Xylose | Optimization of synthetic inoculum conditions in co-cultivation of *E. coli* and *S. cerevisiae* utilizing xylose | Batch | 21.16 mg/L | NA | [12] |

* Percentage yield indicates the ratio of actual yield to theoretical maximum yield expressed in percentage (%).

**Table S2** Naringenin production performance of bacterial strains used in this study.

| **Strain** | **Titer** (mg/L) | **Specific production** (mg/g DCW) | **Yield** | |
| --- | --- | --- | --- | --- |
|  |  |  | **Acetate** (mg NRN/g) | ***p*-Coumaric acid** (g/g) |
| BN | 2.45 | 1.49 | 0.28 | 0.034 |
| BNA | 5.07 | 2.56 | 0.85 | 0.059 |
| BNI | 28.46 | 27.41 | 6.57 | 0.616 |
| BNIA | 32.81 | 29.22 | 6.67 | 0.637 |
| BNIAP100 | 40.67 | 30.97 | 4.41 | 0.577 |
| BNIAP104 | 50.83 | 32.48 | 6.07 | 0.738 |
| BNIAP106 | 56.53 | 64.48 | 7.28 | 0.756 |
| BNIAP115 | 61.23 | 76.60 | 8.43 | 0.759 |
| BNIAP109 | 66.59 | 81.48 | 10.71 | 0.841 |
| BNIAP113 | 61.11 | 71.64 | 10.70 | 0.643 |
| BNIAP103 | 54.33 | 63.74 | 10.48 | 0.553 |
| BNIAP109 (optimized culture condition) | 97.02 | 104.16 | 21.02 | 0.631 |

| **Name** | **Sequence (5′−3′)** |
| --- | --- |
| *acs*_ins_speI_F | cttcctactagtttgacggctagctcagtcctaggtac |
| *acs*_ins_notI_R | gatactgcggccgcgcgcctccatcgagctcaaaaaaaaccc |
| Vector_*acs*_speI_F | cttcctactagtgaagtgcttcatgtggcaggagaaaaaag |
| Vector_*acs*_notI_R | gatactgcggccgcctgtgacggaagatcacttcgcagaataaataaatc |
| *pckA*_UTR_F | caaaactacaaaaggaggatcaaaaatgcgcgttaacaatggtttgac |
| *pckA*_notI_J23115_F | gcatacgcggccgctttatagctagctcagcccttggtacaatgctagccaaaactacaaaaggaggatcaaaaatgcg |
| *pckA*_BBa1006_kpn1_R | gatcgtggtaccaaaaaaaaccccgccctgtcaggggcggggtttttttttttacagcttcggaccagccg |
| Vector_*pckA*_kpnI_F | ctgtacggtacccctggtgtccctgttgataccg |
| Vector_*pckA*_notI_R | gtcacaggcggccgc |
| J23104_*pckA*_blunt_F | ttgacagctagctcagtcctaggtattgtgctagccaaaactacaaaaggaggatcaaaaatgcgcgttaacaatggtttgac |
| J23106_*pckA*_blunt_F | tttacggctagctcagtcctaggtatagtgctagccaaaactacaaaaggaggatcaaaaatgcgcgttaacaatggtttgac |
| J23109_*pckA*_blunt_F | tttacagctagctcagtcctagggactgtgctagccaaaactacaaaaggaggatcaaaaatgcgcgttaacaatggtttgac |
| J23113_*pckA*_blunt_F | ctgatggctagctcagtcctagggattatgctagccaaaactacaaaaggaggatcaaaaatgcgcgttaacaatggtttgac |
| J23103_*pckA*_blunt_F | ctgatagctagctcagtcctagggattatgctagccaaaactacaaaaggaggatcaaaaatgcgcgttaacaatggtttgac |
| *pckA*_blunt_R | ccgcgcgcctccatc |

**Table S3.** Oligonucleotides used in this study.

| **Name** | **Description** | **Source** |
| --- | --- | --- |
| **Strains** | | |
| Mach-T1^R^ | F^-^ φ80(*lac*Z)ΔM15 Δ*lac*X74 *hsd*R(r_K_^-^m_K_^+^) Δ*rec*A1398 *end*A1 *ton*A | Invitrogen |
| BL21star^TM^(DE3) | F^−^ *ompT* *gal* *dcm rne131* *lon hsdS*_B_ (r_B_^-^m_B_^-^ ) λ(DE3) | Invitrogen |
| BL21star^TM^(DE3) Δ*sucC*Δ*fumC* | BL21star^TM^(DE3) Δ*fumC*::*FRTΔsucC*::*FRT* | [4] |
| BN | BL21star^TM^(DE3) / pFlavo^opt^ | This study |
| BNA | BL21star^TM^(DE3) / pFlavo^opt^ / pACYCA | This study |
| BNI | BL21star^TM^(DE3) Δ*iclR* / pFlavo^opt^ | This study |
| BNIA | BL21star^TM^(DE3) Δ*iclR* / pFlavo^opt^ / pACYCA | This study |
| BNIAP100 | BL21star^TM^(DE3) Δ*iclR* / pFlavo^opt^ / pACYCAP100 | This study |
| BNIAP104 | BL21star^TM^(DE3) Δ*iclR* / pFlavo^opt^ / pACYCAP104 | This study |
| BNIAP106 | BL21star^TM^(DE3) Δ*iclR* / pFlavo^opt^ / pACYCAP106 | This study |
| BNIAP115 | BL21star^TM^(DE3) Δ*iclR* / pFlavo^opt^ / pACYCAP115 | This study |
| BNIAP109 | BL21star^TM^(DE3) Δ*iclR* / pFlavo^opt^ / pACYCAP109 | This study |
| BNIAP113 | BL21star^TM^(DE3) Δ*iclR* / pFlavo^opt^ / pACYCAP113 | This study |
| BNIAP103 | BL21star^TM^(DE3) Δ*iclR* / pFlavo^opt^ / pACYCAP103 | This study |
| **Plasmids** | | |
| pKD46 | Red recombinase expression vector, Amp^R^ | [13] |
| pCP20 | FLP expression vector, Amp^R^, Cm^R^ | [13] |
| pETM6 | ColE1 ori, Amp^R^, ePathBrick expression vector | [4] |
| pFlavo^opt^ | pETM6-P_T7_-At4CL-P_T7_-PhCHS-P_T7(C4)_-CmCHI | [4] |
| pACYCduet-1 | p15A ori, Cm^R^, *E. coli* expression vector | Novagen |
| pACYCA | p15A ori, Cm^R^, P_BBa_J23100_-synUTR_acs_-*acs*-Ter_BBa_B1006_ | This study |
| pACYCAP100 | pACYCA-P_BBa_J23100_-synUTR_pck_-*pck*-Ter_BBa_B1006_ | This study |
| pACYCAP104 | pACYCA-P_BBa_J23104_-synUTR_pck_-*pck*-Ter_BBa_B1006_ | This study |
| pACYCAP106 | pACYCA-P_BBa_J23106_-synUTR_pck_-*pck*-Ter_BBa_B1006_ | This study |
| pACYCAP115 | pACYCA-P_BBa_J23115_-synUTR_pck_-*pck*-Ter_BBa_B1006_ | This study |
| pACYCAP109 | pACYCA-P_BBa_J23109_-synUTR_pck_-*pck*-Ter_BBa_B1006_ | This study |
| pACYCAP113 | pACYCA-P_BBa_J23113_-synUTR_pck_-*pck*-Ter_BBa_B1006_ | This study |
| pACYCAP103 | pACYCA-P_BBa_J23103_-synUTR_pck_-*pck*-Ter_BBa_B1006_ | This study |

**Table S4.** Bacterial strains and plasmids used in this study.

**Table S5.** Synthetic 5′ untranslated region sequences and their predicted expression levels.

| **Name** | **5′-UTR Sequence (5′−3′)** | **Predicted**  **expression level^a^**  **(a.u.)** |
| --- | --- | --- |
| *acs* | AAAATCAGCGCCCAAGGAGTCACCG | 1074836.02 |
| *pck* | CAAAACTACAAAAGGAGGATCAAAA | 9348010.52 |

^a^Arbitrary unit of the expression [14]

**Supplementary Figures**


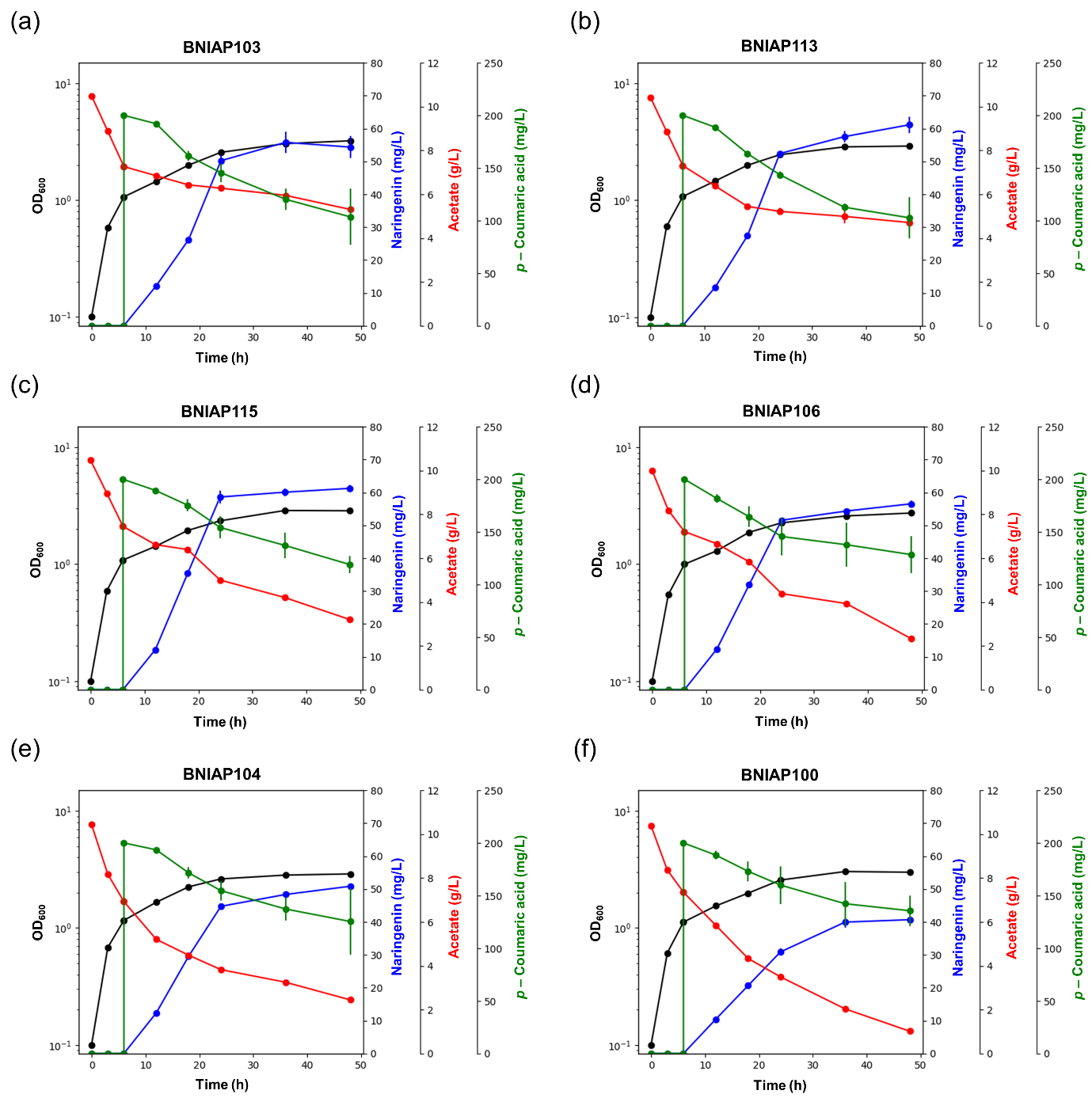


**Fig. S1.** Time-course culture profiles of *pckA* upregulated strain. **(a) – (f)** Culture profiles of *pckA* upregulated variants in various transcriptional levels under different constitutive promoters of J23103, J23113, J23115, J23106, J23104 and J23100, respectively. 200 mg/L *p*-coumaric acid and 1 mM IPTG were added when culture broths reached an OD_600_ of 1.0. BNIAP103, BNIAP113, BNIAP115, BNIAP106, BNIAP104, BNIAP100 refer *Escherichia coli* BL21 Star™(DE3) with heterologous expression of essential enzymes for naringenin production, *acs* overexpression, *iclR* knockout*,* and *pckA* upregulation under constitutive promoter BBa_J23103, BBa_J23113, BBa_J23115, BBa_J23106, BBa_J23104, BBa_J23100, respectively; IPTG, isopropyl β-d-thiogalactopyranoside.

**
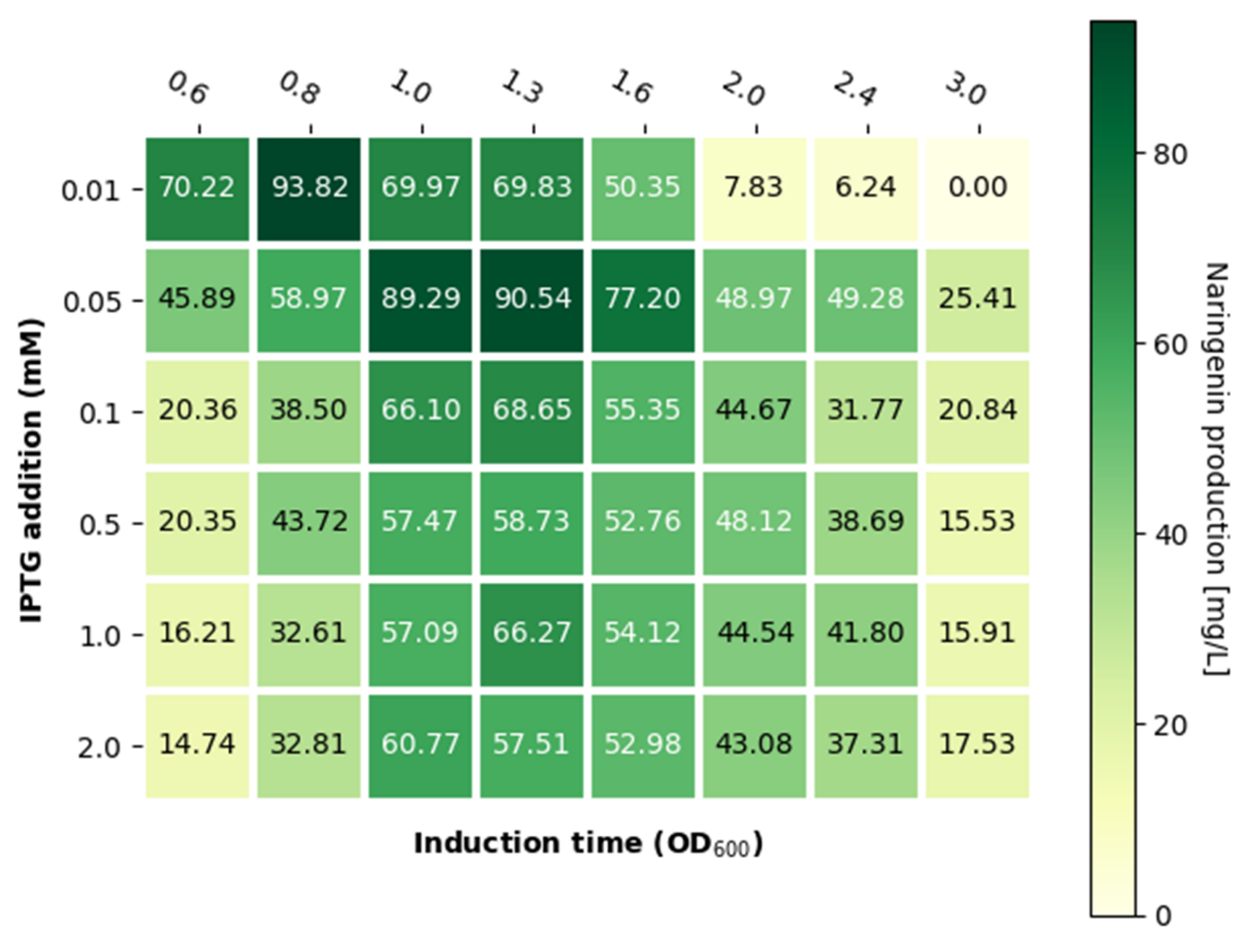
**

**Fig. S2.** Fermentation condition optimization of the BNIAP109 strain in variance of induction time and IPTG concentration. Optimal culture condition was investigated with a variance of IPTG addition in range of 0.01 to 2.0 mM and induction time in series of OD_600_ of 0.6 to 3.0. BNIAP109 refers *Escherichia coli* BL21 Star™(DE3) with heterologous expression of essential enzymes for naringenin production, *acs* overexpression, *iclR* knockout*,* and *pckA* upregulation under constitutive promoter BBa_J23109; IPTG, isopropyl β-d-thiogalactopyranoside.

**
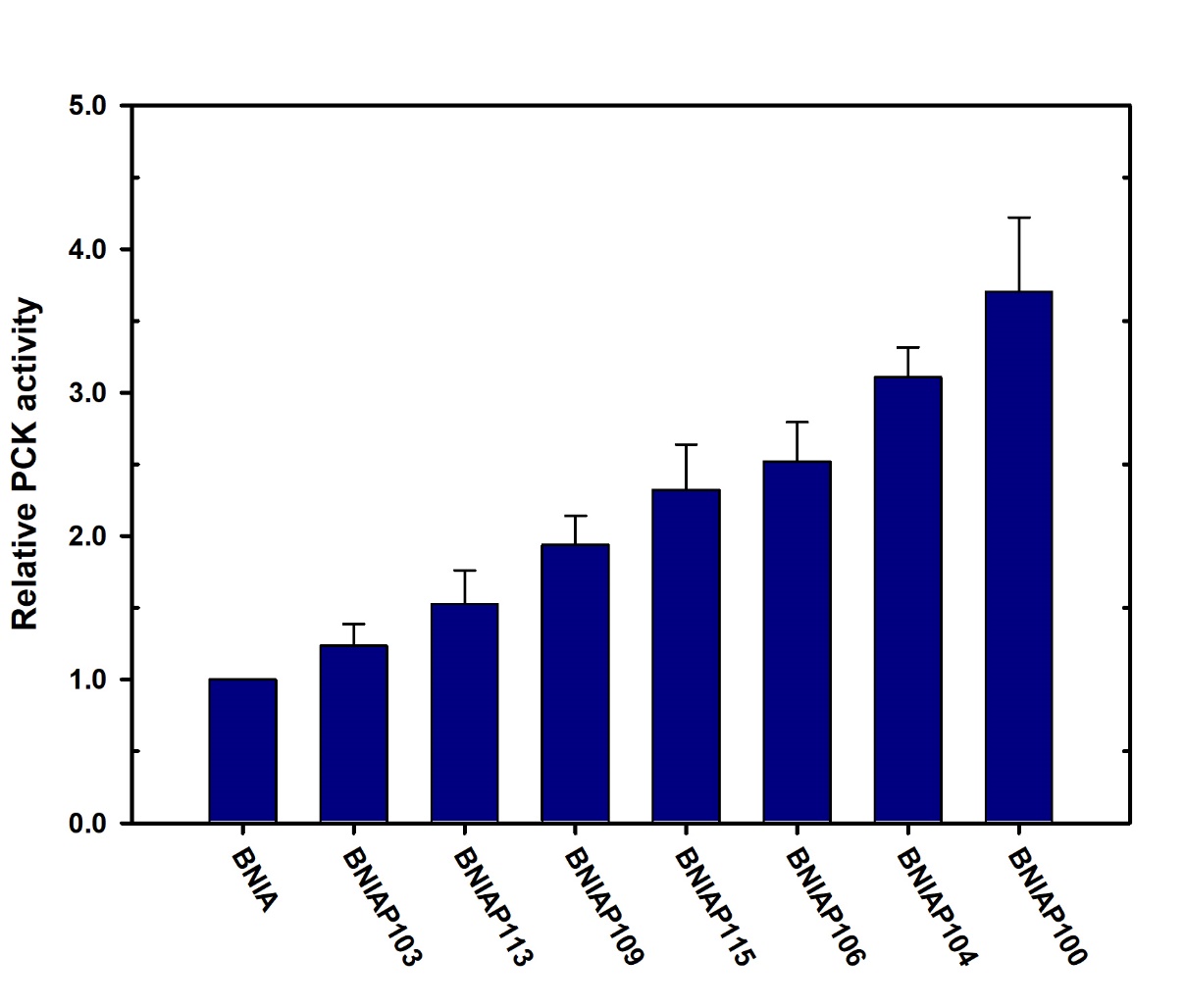
**

**Fig. S3.** Normalized expression level of *pckA* under various promoter strengths. The activity of phosphoenolpyruvate carboxykinase (PCK) was evaluated through the consumption of oxaloacetate for 60 min enzyme reaction at 30 °C. Error bars indicate the standard deviations of biological triplicates. BNIA refers *Escherichia coli* BL21 Star™(DE3) with heterologous expression of essential enzymes for naringenin production, *acs* overexpression and *iclR* knockout; BNIAP103, BNIAP113, BNIAP109, BNIAP115, BNIAP106, BNIAP104, BNIAP100 refer BNIA strain with *pckA* upregulation under constitutive promoter BBa_J23103, BBa_J23113, BBa_J23109, BBa_J23115, BBa_J23106, BBa_J23104, BBa_J23100, respectively; PEP, phosphoenolpyruvate; TCA cycle, tricarboxylic acid cycle; IPTG, isopropyl β-d-thiogalactopyranoside; PCK, phosphoenolpyruvate carboxykinase.

**References**

1. Xu P, Vansiri A, Bhan N, A. G. Koffas M. ePathBrick: A Synthetic Biology Platform for Engineering Metabolic Pathways in *E. coli*. ACS Synthetic Biology [Internet]. 2012;1:256–66. Available from: https://doi.org/10.1021/sb300016b

2. Koopman F, Beekwilder J, Crimi B, van Houwelingen A, Hall RD, Bosch D, et al. De novo Production of the Flavonoid Naringenin in Engineered *Saccharomyces cerevisiae*. Microbial Cell Factories [Internet]. 2012;11:155. Available from: https://doi.org/10.1186/1475-2859-11-155

3. Wang R, F. Cress B, Yang Z, C. Hordines J, Zhao S, Jung GY, et al. Design and Characterization of Biosensors for the Screening of Modular Assembled Naringenin Biosynthetic Library in *Saccharomyces cerevisiae*. ACS Synthetic Biology [Internet]. American Chemical Society; 2019;8:2121–30. Available from: https://doi.org/10.1021/acssynbio.9b00212

4. Jones JA, Vernacchio VR, Sinkoe AL, Collins SM, Ibrahim MHA, Lachance DM, et al. Experimental and Computational Optimization of an *Escherichia coli* Co-culture for the Efficient Production of Flavonoids. Metabolic Engineering [Internet]. Academic Press; 2016;35:55–63. Available from: https://doi.org/10.1016/j.ymben.2016.01.006

5. Hwang HG, Noh MH, Koffas MAG, Jang S, Jung GY. Multi-level Rebalancing of the Naringenin Pathway using Riboswitch-guided High-throughput Screening. Metabolic Engineering [Internet]. Academic Press; 2021;67:417–27. Available from: https://doi.org/10.1016/j.ymben.2021.08.003

6. Watts KT, Lee C, Schmidt-Dannert C. Exploring Recombinant Flavonoid Biosynthesis in Metabolically Engineered *Escherichia coli*. Chembiochem [Internet]. 2004;5:500–7. Available from: https://doi.org/10.1002/cbic.200300783

7. Yang Y, Lin Y, Li L, Linhardt RJ, Yan Y. Regulating Malonyl-CoA Metabolism via Synthetic Antisense RNAs for Enhanced Biosynthesis of Natural Products. Metabolic Engineering [Internet]. Academic Press; 2015;29:217–26. Available from: https://doi.org/10.1016/j.ymben.2015.03.018

8. Leonard E, Lim KH, Saw P-N, Koffas MAG. Engineering Central Metabolic Pathways for High-level Flavonoid Production in *Escherichia coli*. Applied and Environmental Microbiology [Internet]. American Society for Microbiology; 2007;73:3877–86. Available from: https://doi.org/10.1128/AEM.00200-07

9. Ganesan V, Li Z, Wang X, Zhang H. Heterologous Biosynthesis of Natural Product Naringenin by Co-culture Engineering. Synthetic and Systems Biotechnology [Internet]. Elsevier; 2017;2:236–42. Available from: https://doi.org/10.1016/j.synbio.2017.08.003

10. Dinh C v., Prather KLJ. Development of An Autonomous and Bifunctional Quorum-sensing Circuit for Metabolic Flux Control in Engineered *Escherichia coli*. Proceedings of the National Academy of Sciences [Internet]. Proceedings of the National Academy of Sciences; 2019;116:25562–8. Available from: https://doi.org/10.1073/pnas.1911144116

11. Zhou S, Lyu Y, Li H, Koffas MAG, Zhou J. Fine-tuning the (2S)-Naringenin Synthetic Pathway Using an Iterative High-throughput Balancing Strategy. Biotechnology and Bioengineering [Internet]. John Wiley & Sons, Ltd; 2019;116:1392–404. Available from: https://doi.org/10.1002/bit.26941

12. Zhang W, Liu H, Li X, Liu D, Dong X-T, Li F-F, et al. Production of Naringenin from D-xylose with Co-culture of *E. coli* and *S. cerevisiae*. Engineering in Life Sciences [Internet]. John Wiley & Sons, Ltd; 2017;17:1021–9. Available from: https://doi.org/10.1002/elsc.201700039

13. Datsenko KA, Wanner BL. One-step Inactivation of Chromosomal Genes in *Escherichia coli* K-12 using PCR products. Proceedings of the National Academy of Sciences [Internet]. National Academy of Sciences; 2000;97:6640–5. Available from: https://doi.org/10.1073/pnas.120163297

14. Seo SW, Yang JS, Cho HS, Yang J, Kim SC, Park JM, et al. Predictive Combinatorial Design of mRNA Translation Initiation Regions for Systematic Optimization of Gene Expression Levels. Scientific Reports [Internet]. Nature Publishing Groups; 2014;4:4515. Available from: https://doi.org/10.1038/srep04515
